# Effect analysis of government intervention on scale-heterogeneous farmers’ behavior of groundwater exploitation

**DOI:** 10.1371/journal.pone.0304015

**Published:** 2024-08-12

**Authors:** Xin Wang, Qian Lu, Zhaohua Zhang

**Affiliations:** 1 Department of Economics, Tianjin University of Commerce, Tianjin City, China; 2 Department of Economics and Management, Northwest A&F University, Shannxi City, China; Balochistan University of Information Technology Engineering and Management Sciences, PAKISTAN

## Abstract

Government intervention has become an important measure to restrain groundwater overexploitation. This paper analyzes the effect of three types of government intervention measures, namely, guidance, incentive and constraint, on farmers’ groundwater utilization behavior, from the perspective of scale-heterogeneity, using general quantile regression model, by survey data of 1122 households in well irrigation area of north China. The results showed that: (1) the incentive and guiding measures have negative effects on farmers’ groundwater usage, while the effect of restrictive measures is not obvious. The guided policy is superior to the incentive measure as to governance effect. (2) With the increase of farmers’ land scale, the influence of incentive measures shows a trend of weakening, and the effect of guided measures on groundwater demand reduction of farmers is stronger. When it comes to the different point of water consumption, when at the point level of 0.25, the incentive measures have the most obvious inhibitory effect. With the increase of water consumption of farmers, the guided measures begin to play a core role. The effect of restrictive measures is not obvious with the increase of water consumption. (3) In addition, farmers’ irrigation water consumption also is affected by gender, cognition of water resources shortage, ecological cognitive level, acquisition ability of disaster information, village rain conditions, the degree of water rights market development, feelings of water fee increase, irrigated disputes in the village, collective economic level of village. The selection of policy tools is flexible according to the farmers’ land scale for groundwater over-extraction control.

## Introduction

The North China Well Irrigation District is one of the important food production areas in China, where output of wheat and corn account for more than 50% and 30% of the total national production respectively, and the demand for irrigation water is strong. In order to meet the growing demand for agricultural irrigation water, local farmers have exploited a large amount of groundwater, which has caused over-exploitation of groundwater and a series of ecological degradation and geological environmental problems such as water level decline, backwater irrigation, and land consolidation [1–3], which seriously threatens the security and sustainable development of food production in the North China Well Irrigation District. Groundwater management plays the importance role of ecosystem services [4]. In recent years, governments have adopted a series of intervention measures in order to solve the problem of over-exploitation of groundwater, such as setting water consumption redlines, water price regulation, water-saving subsidies, collection of water resources taxes and fees, laws and regulations on water resources management, education about water conditions, guiding farmers to reduce groundwater extraction and carry out ecological protection. The goal is to ensure the flexibility of individual farmers’ water use and achieve a balance in the total amount of supplements. The effect of the policy is gradually emerging [5–11]. However, as the intervention policy did not fully take into account the heterogeneity of farmers [12], especially with different farmland sizes leads to “difficulty and inefficiency in policy implementation”. [13] Farmers’ overexploitation groundwater behavior is still difficult to stop.

In essence, groundwater is a kind of public good, and easy to cause "tragedy of the commons." According to the theory of public choice, the governance of groundwater over-exploitation requires the government to adopt various measures to intervene and guide farmers’ water behavior, and the response of farmers to government intervention policies directly determines the effectiveness of intervention measures. At present, the academia has paid attention to the fact that groundwater is over-exploited, and has conducted a lot of discussions on the groundwater over-exploitation behavior of farmers. It is generally believed that farmers’ decision-making on irrigation behavior is subject to water resources attitude, values, education, income, irrigation area, water price, water fee management, water rights trading markets, social information, participatory irrigation management, government subsidies, water associations, and village environment [14–20], concluded that farmers’ behaviors are not only constrained by their own needs and resource constraints, but also affected by the socio-economic environment and government policy intervention. Therefore, relying on government departments to intervene in water resources management, especially in arid regions, has become a universally accepted concept for governments around the world. Wang Xiaojun et al. [21] considered that the water resources management measures based on market mechanism-based price adjustment and administrative management-based quantity control in arid regions are important tools for effectively alleviating the contradiction between water supply and demand. Water control is better than water price adjustment to save water. Bate [22] also reached a unanimous conclusion, suggesting that attempts be made to rely on legal, policy and regulatory agency management innovations in water control to achieve effective water resources management. However, different types of regions have different water resource management measures. Some scholars focus their research on groundwater resource management in North China, and believe that the core problem of groundwater over-exploitation is that difficult to supervise and manage groundwater [23]. Encouraging farmers to save water becomes an effective way through supervision and compensation policies. At the same time, strengthening publicity efforts for serious consequences caused by groundwater over-exploitation is also an important measure for government departments to improve the status of groundwater management [24]. Although the national government has issued a number of water resources management policies, especially groundwater resources management policies, due to the typical top-down characteristics of such management policies, there is a lack of attention to water users’ demand response and willingness to participate, and the heterogeneity of water resources conditions and government reform cognition in different regions has resulted in water resources management policies that have not achieved satisfactory results, and there is still a gap between policy goals and water-saving incentives. At the same time, the existing literature focuses on the analysis of factors affecting farmers ’water-saving behaviors and the normative analysis of groundwater resource management policies, ignoring the different farmers’ responses to various types of management policy behaviors, especially the quantitative investigation of groundwater mining behavior responses. However, only by fully understanding the farmers’ responses to different types of policy interventions and the water-saving effects after the implementation of the policies, can they better formulate groundwater resource management policies and effectively solve the problem of groundwater over-exploitation.

In addition, as the government encourages the transfer of rural land, the farmer ‘s cultivated land size has become increasingly differentiated [25], showing heterogeneity, and there is a large difference in the behavior of farmers in different farmland sizes, even if the same farmer has The response behavior exhibited is not completely consistent. However, the current research does not carefully analyze the dynamic impact of government interventions on the groundwater use behavior of large-scale heterogeneous farmers, which is the crux of the policy’s inefficient implementation.

Based on this, this paper uses the survey data of farmers in North China Well Irrigation District to explore the dynamic effects of government interventions on groundwater use behavior of large-scale heterogeneous farmers. Compared with previous studies, this study is unique in that: (1) the government interventions are divided into three types: restraint, incentive, and guidance; (2) using quantile regression models to investigate the dynamic effects of interventions on groundwater use behavior of farmers with different farmland scales, and to provide an empirical basis for the government to formulate differentiated intervention policies. A comprehensive study and evaluation can not only examine the effectiveness of government intervention policies, but also discuss how to take targeted measures according to farmers’ scale heterogeneity in the operation of irrigation overexploitation control policies, and explore paths and accumulation for the implementation of national groundwater overexploitation control policies experience.

## Empirical observation and theoretical framework

### Empirical observation and classification of government interventions

According to the theory of public choice, government intervention is required in order to avoid market failure. Government intervention includes a series of measures that regulate and correct the behavior of subjects. In order to effectively control over-exploitation of groundwater, the government has successively carried out pilot projects for comprehensive management of over-exploitation of groundwater in Hebei and other places, strengthened the government’s function of controlling water withdrawal, and strictly managed water resources. Specific manifestations include the formulation of a series of measures such as the regulations on groundwater management, the opinions on the implementation of the most strict water resources management system, the comprehensive governance policy documents for groundwater overdraft, the provision of red lines for groundwater resources in groundwater overdraft areas, and the usage of dynamic real-time monitoring water level and water volume platform in order to control water withdrawal, and implement the most stringent water resources management system. Some regional governments check the water allocation of farmers based on crop water quotas, planting structures, arable areas, etc., issue water rights certificates, and issue "implementation opinions on promoting comprehensive reform of agricultural water prices", "agricultural water price reforms and reward measures" to achieve agricultural water use. Households use water within the water rights quota to charge for parity water. Less water consumption within the quota is rewarded. Water that exceeds the water quota is charged for high-priced water. A precise subsidy for agricultural water prices and a water-saving reward system have been established. In addition, by making full use of various media and using "World Water Day" and "China Water Week", water laws, water regulations, water rights, structural water conservation, agronomic water conservation, engineering water conservation, and other water conservation policies and technologies have been fully implemented. Publicity and training in various aspects to raise farmers’ awareness through the distribution of leaflets, brochures, hanging banners, etc., to comprehensively create a good atmosphere for water conservation and water conservation, further increase farmers’ awareness of water conservation and promote the enthusiasm and consciousness of applying water-saving technologies. Although these measures have achieved some results, due to their top-down characteristics and lack of feedback on farmers’ adaptive behavior responses, the implementation effects of different types of measures are uneven, and the contradiction of over-exploitation of groundwater is still outstanding. Based on this, this paper divides government interventions into three types of restraint, incentive and guidance through the practice of rural surveys and discussions with relevant scholars in agricultural economics and hydraulics.

Restrictive policy refers to the government’s control of farmers’ groundwater behavior by relying on laws, regulations, rules, administrative method. It aims to regulate farmer household behaviors by using punitive measures such as setting water red lines, shutting down private excavator wells, and controlling irrigation water consumption in groundwater over-exploitation areas. This type of measures has a compulsory meaning. When farmers overexploit, they will face external overexploitation costs caused by the divergence of internalized individual costs and social governance costs. At the same time, the government also needs to bear high monitoring and enforcement costs.

Incentive policy refers to the government’s use of water price adjustment, water saving incentives and other methods to control over-exploitation of groundwater. The purpose is to make farmers gain extra income through price increases or rewards. This type of measure assumes that farmers’ income is maximized, and changes the farmers’ income function through economic incentives. Farmers make reasonable choices after comparing the costs and benefits. However, due to the heterogeneous characteristics of different farmers, their income functions are different, which leads to the uncertainty of the governance effect of this economic measure on different individuals [26, 27].

Guided policy refers to the government’s promotion and education of water saving technology training and groundwater treatment to farmers. Such measures serve as a "soft tool" to guide the communication method, arouse the consciousness of groundwater over-exploitation management of farmers, stimulate the consciousness and initiative of public participation of farmers, and help farmers join in public welfare activities for over-exploitation management of groundwater [28]. On the one hand, after participating in training or being educated, farmers change their perceptions and save water. On the other hand, this kind of guided measures is easy to form a community norm after it is widely adopted in the community. This kind of water-saving social norms will stimulate farmers’ enthusiasm for supervising underground water extraction and complying with water conservation commitments.

### Theoretical framework

Groundwater resources are typical public products. What are the mechanisms of different types of interventions for over-exploitation of groundwater? What is the difference under different scale conditions? It is an urgently needed question to control groundwater over-exploitation. This section explores the mechanism of the impact of restraint, incentive, and guide farmers on groundwater use behavior of farmers, and builds a theoretical framework for research.

### Impact of constrained measures on groundwater utilization behaviors of farmers

Restrictive measures use mandatory administrative orders to restrict farmer behavior. Groundwater is a public resource and has non-exclusive characteristics, so a large number of farmers have been exploiting groundwater. However, the ecological hazards and food security hazards caused by overexploitation of groundwater threaten the overall social welfare level. The government uses strict penalties to restrict over-exploitation of groundwater by farmers. The ultimate goal is to control over-exploitation of groundwater to maximize social welfare and achieve the "Lindall equilibrium." The specific path is to implement a strict water resource management system to supervise the over-exploitation of groundwater by farmers. When violations of the management red line are punished, increase the private cost of over-exploitation of groundwater by farmers. In order to minimize the private costs, farmers are willing to reduce groundwater use and reduce the high costs caused by the implementation of restrictive measures [29].

### Impact of incentive measures on groundwater use behavior of farmers

Incentive measures control the use of groundwater by subsidizing farmers’ groundwater use or price regulation. It is essentially an economic incentive, that is, an exogenous price is added to the groundwater price, and the price signal released will affect the farmers’ cost-benefit expectations, and then change the farmers’ behavior function. Incentive measures are assumed to maximize farmers’ income, and adjust farmers’ behavior mainly from the perspective of farmers’ income [30]. The specific path is to influence the farmer’s income function according to the farmer’s water saving subsidy or water price regulation. In order to maximize personal income, farmers are willing to reduce the use of groundwater and obtain additional benefits from subsidies or price regulation.

### Impact of guiding measures on groundwater utilization behaviors of farmers

The purpose of the guidance measures is to create an atmosphere for water saving for farmers, and to change their perception of water saving through subtle influences. Essentially, it interferes with farmers’ psychology through signal transmission [31, 32], empowers the public to save water (Bowles, 2008), and enhances their awareness of rules. The government has extensively publicized the dangers of groundwater over-exploitation and the trend of water shortage, passed the important information to farmers that groundwater over-exploitation seriously threatened the sustainable development of agriculture, and created a goodwater-saving atmosphere and social norms, thereby changing farmers’ attitude of water resources farmers will use groundwater more cautiously and reduce groundwater extraction.

**Fig 1:**
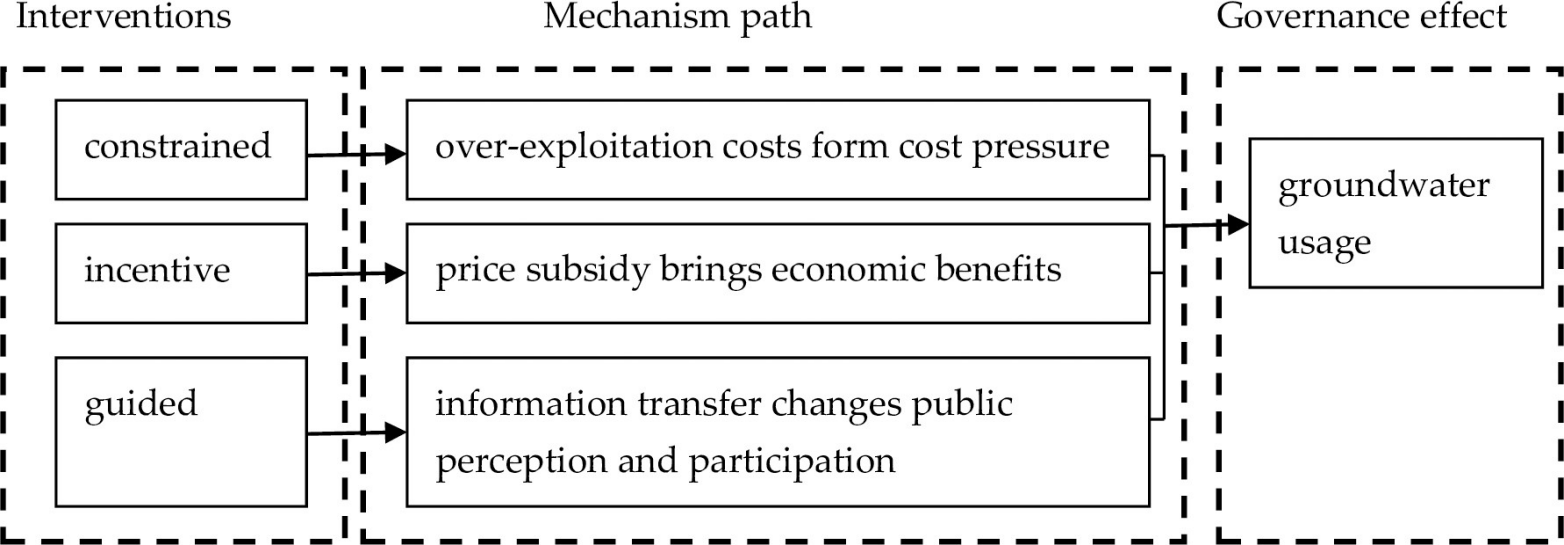
The mechanism path of interventions’ governance effect on groundwater usage. In the left dotted box shows three interventions including constrained, incentive and guided interventions. In the middle dotted box shows three interventions’ mechanism path of groundwater usage. In the right dotted box shows the governance effect which is measured by groundwater usage.

In summary, restrictive measures increase farmers’ over-exploitation costs through policy implementation, incentive measures bring economic benefits through marketization (price increases or subsidies), and guided measures change water awareness and public affairs participation through signal transmission, and ultimately affect farmers’ water behavior. The mechanism path of interventions’ governance effect on groundwater usage is shown in Fig 1. The difference in farmland size of farming households makes the effects of different types of heterogeneous interventions.

## Model building and methodology

This paper first uses a general regression model to estimate the overall impact of different types of interventions on groundwater extraction behavior of farmers. The specific model is as follows.

$$y_{i}(x_{i})=x_{i}^{,}\beta_{i}+\varepsilon$$

y_i_ represents the water consumption of the i-th farmer, x_i_ represents the matrix set of different variables of the i-th farmer, β_i_ is the regression coefficient, and ε is the random interference term.

However, due to the differences in the effects of government interventions in different groundwater distribution situations, the general regression model cannot accurately grasp the impact of government interventions on the distribution of groundwater mining behavior. The methodology of this study is quantile regression model, compared with the least squares method, which breaks through the strict assumption limit, can effectively capture the distribution of multiple quantiles of the dependent variable, and accurately characterize the variation range of the independent variable’s influence on the dependent variable, making the estimation excellent and robust. Therefore, in order to elaborate the impact of different government interventions on groundwater extraction, a quantile regression model is selected for estimation.

Assume that Y is a random continuous variable, that is, the amount of groundwater used by the farmer, and F_y_(y_q_) is the cumulative distribution function. The quantile function model isas follows.

$$y_{q}(x_{i})=x_{i}^{,}\beta_{q}$$

Where y_q_ represents the q quantile of y, which are 0.25, 0.5, and 0.75, respectively, and satisfy $q=P(Y\leq y_{q})={F_{y}(y}_{q})$. x_i_ represents the matrix set of different variables of the i-th farmer. β_q_ is the "q quantile regression coefficient", which measures the degree of influence of individual and household variables, economic environment variables, village characteristics, and government intervention measures on the distribution of groundwater use by farmers. The calculation of quantile regression estimators is based on an asymmetric form of absolute residual minimization using Least Absolute Deviations estimation Estimator (LAD).The formula for calculating the estimated value is as follows.


$$\underset{\beta_{q}}{\min}\sum_{i:y_{i}{\geq x}_{i}^{,}\beta_{q}}^{n}q|y_{i}{-x}_{i}^{,}\beta_{q}|+\sum_{i:y_{i}{<x}_{i}^{,}\beta_{q}}^{n}\left(1-q)|y_{i}{-x}_{i}^{,}\beta_{q}\right|$$

## Data collection and statistics

### Overview of the study area

In order to elaborate the internal mechanism of the effect of government intervention on groundwater utilization of different farmers, this paper selects the North China Well Irrigation District, which is caused by geographical and natural climatic conditions, and has a prominent groundwater over-exploitation problem. The North China Well Irrigation District is located in the North China Plain. The climate belongs to a temperate continental climate. The average annual rainfall is 400-500mm and the average annual evaporation is under 1600mm [32]. It belongs to a typical semi-arid and water-deficient region. The region’s food crops are mainly wheat, corn, and soybeans. Because the North China Well Irrigation District has undertaken more than a quarter of China’s food production, farmland irrigation requires a large amount of water resources. In order to meet the growing demand for irrigation water, local farmers have developed large-scale groundwater, and the number of groundwater wells has increased rapidly. According to statistics from provinces and municipalities in North China, as of the end of 2005, the number of machine wells was as high as 3.743 million, and the total groundwater mining volume exceeded 40 billion cubic meters, accounting for 30% of the national mining volume [24] Excessive groundwater mining caused the ground to collapse, forming a huge funnel area in the North China Plain, with an area of more than 8,800 square kilometers. The shortage of groundwater resources has led to the deepening of the farmer’s machine wells, and the phenomenon of overexploitation of groundwater has become increasingly prominent, forming a vicious cycle of "water shortage-mining-excessive water shortage-overexploitation", which seriously threatens local food security and sustainable agricultural development.

### Data sources

The data are mainly from the field survey conducted by the Tianjin Water Resources Research Team in January 2021. In the design of the survey plan, the survey was conducted in six typical areas: TaoCounty, Hengshui, Hebei Province; Wuqiao County, Cangzhou City, Hebei Province; Ningling County, Shangqiu City, Henan Province; Feicheng County, Tai’an City, Shandong Province; Wuqing District in Tianjin, and Miyun District in Beijing. These areas belong to over-exploitation of groundwater areas in the North China Well Irrigation District listed by Local Water Agencies. This paper’s survey was conducted by using random sampling method based on implicit stratification (stratification indicators are region, irrigation attributes and per capita GDP). In the first stage, villages were ranked in terms of rural irrigation attributes and per capita GDP and then randomly selected in each selected county or district unit with proportional to population size probability. In the second stage, all the households, whose age between 20 and 80 listed by village committee, were randomly selected in each selected village with proportional to population size probability and all the samples includes total of 1,200 farm households. The sampling weight is the reciprocal of the sampling probability, which is proportional to population size probability, at each sampling stage during the sampling process. The survey used face-to-face random interviews, and the respondents were mainly heads of households who are able to communicate normally, engaged in agricultural production and management, and have strong decision-making abilities. According to rules of selecting sample sizes, the confidence interval is 95% with an allowable error of 3% and the sample size is 1067. So our sample sizes are suitable. The content of the survey is mainly focused on issues such as groundwater utilization and government intervention. In order to ensure the accuracy of the questionnaire survey, a preliminary survey was conducted, and after the survey, the questionnaire was adjusted according to the situation and a formal survey was conducted. The number of questionnaires finally obtained was 1122 excluding questionnaires with incomplete or illogical answers, and the effective rate was 93.5%. Table 1 shows the questionnaire data from the surveyed sample.

**Table 1:** Variable description and descriptive statistics of the survey sample.

Variables	name	definition	means	S.D.	min	max
Explained variable						
	Annual average groundwater use	Annual average groundwater use (Cubic meters / mu) logarithmic	4.843	1.134	1.522	8.178
Government Intervention Measure						
	Restrictive measure	Are you supervised for groundwater overexploitation? Or administrative penalties? Strongly disagree = 1, more disagree = 2, average = 3, more agree = 4, strongly agree = 5	2.485	1.296	1	5
	Incentive measures	Have you ever received water-saving subsidies or price incentives? Strongly disagree = 1, more disagree = 2, average = 3, more agree = 4, strongly agree = 5	1.701	1.385	1	5
	Guided measures	Have you received government training or promotional information on water-saving or groundwater over-exploitation hazards? Strongly disagree = 1, more disagree = 2, average = 3, more agree = 4, strongly agree = 5	3.693	0.867	1	5
Other control variables						
Household And family Characteristics	Age	Under 35 = 1, [35–50] = 2, [50–65] = 3, [65–80] = 4	3.077	0.706	1	4
	Gender	male = 1, female = 0	0.549	0.498	0	1
	Education level	Illiteracy = 1; primary school = 2; junior high school = 3; high school and technical school = 4; junior college and above = 5	2.363	1.261	1	5
	Water shortage Cognitive level	Do you think there will be a crisis of water shortage in the future? Strongly disagree = 1, more disagree = 2, average = 3, more agree = 4, strongly agree = 5	2.679	1.142	1	5
	Ecological cognition level	Does over-exploitation of groundwater harm ecological environment? Yes = 1, unclear = 2, no = 3	2.009	0.578	1	3
	Disaster information acquisition capability	Your ability to acquire information on disasters such as droughts and floods is strong? Strongly disagree = 1, more disagree = 2, average = 3, more agree = 4, strongly agree = 5	3.401	1.257	1	5
	Farming ratio	Percentage of long-term agricultural labor to family labor	0.409	0.315	0	1
Economic environment	Development of water rights market	What’s the level of development of water rights market? Degree Unsound = 1, More healthy = 2, Very healthy = 3	1.636	0.483	1	3
	Feelings of rising water prices	In the past three years, the price of water has increased significantly? Strongly disagree = 1, more disagree = 2, average = 3, more agree = 4, strongly agree = 5	3.078	1.118	1	5
Village characteristics	Village rain conditions	What is the rain condition of your village in the past three years? Frequent rain = 1, occasional rain = 2, droughts = 3	2.139	0.602	1	3
	Village water disputes	Do you often have water disputes in your village during the busy season? No dispute = 1, occasional = 2, frequent = 3, very frequent = 4	2.251	1.216	1	4
	Village collective economic level	Is your village’s collective economy more developed? Strongly disagree = 1, more disagree = 2, average = 3, more agree = 4, strongly agree = 5	1.844	1.249	1	5

### Variable selection and descriptive statistics

Because groundwater extraction measurement, especially the measurement of groundwater extraction of farmers, has no consistent conclusion, and the measurement is difficult, this article combines the relevant literature and field survey data to use the average annual groundwater irrigation amount of farmers as groundwater extraction. The measured index, which is the explanatory variable, is set to the question "How many cubic meters of water do you use for machine irrigation per year?" It is verified based on the irrigation quota and water well electricity consumption method. Divide the independent variables into core independent variables and other control variables. The specific explanation and descriptive statistics are as follows.

### Core independent variables: Government intervention

According to the current groundwater resource management policies implemented by the government, government interventions are divided into three types: restrictive, incentive, and guidance. The question of obtaining the three types of data set in the questionnaire is: "Are you supervised for groundwater overexploitation? Or administrative penalties? "" Have you ever received water-saving subsidies or price incentives? "" Have you received government training or promotional information on water-saving or groundwater over-exploitation hazards? "

From the perspective of different types of enforcement efforts (see Table 1), the average values of incentive measures, guidance measures, and restraint measures were 1.701, 3.693, and 2.485 respectively. There are more publicity and education on mining hazards; followed by restrictive measures, farmers have been subject to regulation or administrative punishment for groundwater over-exploitation. Finally, incentive measures, farmers have less access to subsidies or water price regulation incentives, although the state, regional governments and grassroots township organizations have implemented various subsidies and incentive measures, but farmers benefit less.

### Other control variables

This paper also sets the characteristics of individual households and families, economic environment variables and village variables as control variables. In terms of individual heads of households and household characteristics, the majority of the heads of the households surveyed are between 50 and 65; males account for 54.9%; education levels are concentrated in primary school education; farmers’ awareness of the crisis of water shortages in the future and their overreach to groundwater. The consciousness of mining harming the ecological environment is relatively weak. Most farmers are more optimistic about the future supply of water resources, and it is not clear that over-exploitation of groundwater will harm the ecological environment. The information acquisition ability of household heads in droughts, floods and other disasters is moderately high. The average level of the labor force engaged in agricultural production to the household labor force was 0.416. In terms of economic environmental variables, farmers believe that the local water rights market is not sound and that their feelings about rising water prices are not obvious. In terms of village characteristics, there have been occasional droughts or floods in the villages, and there have been fewer disputes over water use, and the village’s collective economy is not sufficiently developed.

## Results and discussion

### Impact of government intervention on groundwater use behavior of the overall sample farmers

This section uses Stata 14.0 statistical software and uses a general regression model to perform a regression analysis on the overall sample. Table 2 is obtained. The model passes the 1% significance level test. The specific interpretation results are as follows. Jackknife estimation is used in the model to avoid bias and R^2^equals 0.3345.

**Table 2:** Empirical results of government intervention on groundwater use of farmers.

Groundwater extraction	coefficient	T-value	P-value
Core variable: government intervention			
Restrictive measures	-0.015	-0.96	0.492
Incentive measures	-0.162***	-6.37	0
Guided measures	-0.13***	-4.82	0
Other control variables			
Age	-0.01	0.45	0.819
Gender	0.101*	2.45	0.084
Education level	-0.024	5.18	0.3
Water shortage awareness level	-0.136***	-3.8	0
Ecological Cognitive Level	-0.17***	-3.62	0.003
Disaster information acquisition capacity	0.051**	1.38	0.022
Farming ratio	-0.223**	-1.02	0.023
Development degree of water rights market	-0.351***	-3.63	0
Feeling of rising water prices	-0.027	-1.56	0.285
Village rain conditions	-0.175***	-0.8	0.003
Village water dispute	0.307***	10.42	0
Village collective economic level	0.163***	6.95	0
Constant term	5.979***	19.01	0

Note

***, **, * indicate significance levels of 1%, 5%, and 10%, respectively.

### Government intervention

The incentive and guidance measures passed the 1% significance test, respectively, and the signs were negative, that is, the stronger the implementation of the incentive and guidance measures, the more obvious the effect of reducing the amount of groundwater irrigation for farmers. The coefficients of restrictive, incentive and guided measures are -0.015,-0.162,and -0.130, respectively. This shows that the degree of impact of three different policy measures on farmers’ groundwater use is as follows, the guidance is better than the incentive and the constraint. The difference between the guiding effect and the incentive effect is small, while the effect of restraint measures is not obvious. In terms of reducing mining, farmers are more willing to accept guidance measures with soft constraints, such as publicity. According to the theory of attitude and behavior, guidance will change personal attitudes, and the promotion of water-saving policies and other content can create a water-saving atmosphere, affect farmers’ cognition through subtle ways, and strengthen farmers’ water-saving willingness and attitudes. Farmers will treat water resources more cherishly and reduce water consumption. This also validates Wang Hui’s [26] opinion that guidance tools will take precedence over other policy tools. Incentive measures are mainly rewards and subsidies for water conservation. When farmers use water-saving measures, water-saving incentives or subsidies can make up for the cost and loss of water consumption by farmers, or even bring in additional income such as income and prestige due to subsidies or incentives. Based on the assumption of a rational economic person, farmers will tend to reduce water consumption. Restrictive measures are typical command-and-control policies. They force farmers to reduce water consumption by means of compulsory measures. Due to the emergence of rent-seeking and free-riding behaviors, the restrictive water-saving effect is not obvious. If it is not performed properly, it can even lead to opposing emotions. In the process of policy implementation, it is faced with a high cost of policy implementation and supervision, resulting in insufficient supervision by supervisors. At the same time, because farmers are scattered, rational farmers with the goal of minimizing private costs will choose opportunistic methods to avoid penalties, resulting in excessive implementation costs of restrictive measures [33, 34]. The final result was that the restrictive measures caused institutional failures in the treatment of farmers’ groundwater over-exploitation. The survey also found that although the surveyed farmers’ perception of the implementation of restraint measures is at a general level, which is higher than that of incentive measures, the restraint measures have not exerted a restraining effect on farmers’ groundwater overexploitation. The empirical results show that in terms of controlling over-exploitation of groundwater, the measures more easily accepted by farmers are the kind of dazzling propaganda and education-oriented methods, and the failure to respond to compulsory measures.

### Other control variables

Gender is at the significance test level of 10%, and the coefficient is positive, which indicates that gender has a significant positive impact on farmers’ increase of groundwater irrigation. Men are more inclined to increase water use. Males are not sensitive for the cost of irrigation and water-saving technology compared with females [34]. The water scarcity awareness level passed the significance level test of 1%, and the direction is negative, that is, the higher the awareness level, the more willing farmers are to reduce groundwater irrigation. The level of awareness of water shortage measures the level of awareness of farmers on whether there is a crisis of water shortage. The higher the level of awareness, the more willing the farmers are to improve the current excessive use behavior and avoid water waste [35]. The level of ecological cognition passed the significance level test of 1%, and the coefficient was negative, which indicates that the higher the level of ecological cognition, the more farmers prefer to reduce groundwater use. The ecological cognition level measures the degree of farmers’ awareness of the harm caused by groundwater overexploitation. The higher the level of awareness, the more willing the farmers to use groundwater cautiously [36]. This also validates the core idea of planned behavior theory, that individual behavior is affected by cognitive level [37]. Farm ratio has passed the significance level test of 1% and has a negative direction. This shows that when more farmers pay more attention to the farming, the less water amount they use. Farmer households with higher farming ratio have accumulated rich irrigation water experience due to engaged in agricultural production activities for a long time, and are more inclined to apply water-saving measures to reduce irrigation water use.

The development level of the water right market has passed the significance level test of 1% and has a negative direction. This shows that the more sound the water right market is, the more willing farmers are to save groundwater, which is consistent with the views of Liu Yiming and Luo Biliang [38]. When the water rights market operates soundly, farmers tend to place the saved water consumption in the water rights market for trading to obtain certain economic benefits. Rising water prices have little effect on farmers’ water use.

Village rain conditions has passed the significance level test of 1% and has a negative direction. The abundance of rain in a village has a great influence on the amount of water used for irrigation. The more rain there is, the less water used for irrigation.

Village water disputes have passed the 1% significance test, and the direction is positive. This shows that the more frequent village water disputes, the more willing farmers are to chase groundwater, which is related to the farmers’ profit-seeking psychology and the attributes of groundwater public goods [39]. The village collective economic level passed the significance test of 1%, and the sign was positive. From this, we can see that the more developed the village collective economy, the more severe the over-exploitation of groundwater for farmers. The results of this analysis warn to avoid the serious consequences of over-exploitation of groundwater and waste of water resources while expanding the economic development of villages, especially in the context of the current rural revitalization, and to attach great importance to rural water resources management.

### Impact of government intervention on groundwater use of farmers with different farmland scales

This section uses 5 acres as the cutoff point, and performs quantile regression on samples above (0,5), (5,10), and 10 acres. Discuss the impact of government interventions on groundwater use behavior of farmers with different farmland scales. For the convenience of statistics, the quantiles are estimated at 0.25, 0.5, and 0.75. The statistical results are shown in Table 3.

**Table 3:** Quantile regression results of the effects of government interventions on groundwater use behavior of farmers with different farmland sizes.

	(0,5) N = 496					
	q25	P-value	q50	P-value	q75	P-value
Restrictive measure	-0.0565	0.212	-0.0511	0.258	-0.0149	0.763
Incentive measures	-0.248***	0	-0.132***	0.007	-0.188***	0
Guided measures	-0.0595	0.371	-0.0896	0.178	-0.0824	0.256
agelevel	-0.0659	0.442	-0.0381	0.656	0.0117	0.9
gender	0.174	0.138	0.00704	0.952	0.0557	0.663
edulevel	-0.0695	0.161	-0.0517	0.297	-0.0676	0.211
Water shortage cognitive level	-0.188***	0	-0.136***	0.01	-0.0791	0.172
Ecological cognition level	0.0936	0.414	0.309***	0.007	0.166	0.183
Disaster information	0.0130	0.799	-0.0617	0.227	0.0524	0.346
Farm ratio	-0.222	0.22	-0.108	0.55	-0.0289	0.883
Development of water rights market	-0.587***	0	-0.179	0.203	-0.172	0.263
Feelings of rising water prices	-0.0412	0.474	-0.0668	0.245	0.0226	0.718
Village rain conditions	-0.0759	0.473	-0.0167	0.874	-0.0494	0.668
Village water disputes	0.208***	0	0.388***	0	0.329***	0
Village collective economic level	0.186***	0.001	0.0372	0.51	0.0813	0.186
_cons	5.734***	0	5.404***	0	5.382***	0
	(5,10) N = 520					
	q25	P-value	q50	P-value	q75	P-value
Restrictive measure	-0.0307	0.404	0.0277	0.442	0.0478	0.294
Incentive measures	-0.171***	0	-0.169***	0	-0.168***	0.001
Guided measures	-0.165***	0.003	-0.205***	0	-0.203***	0.003
agelevel	-0.138**	0.045	-0.0159	0.813	-0.00182	0.983
gender	0.129	0.176	0.148	0.113	-0.0137	0.907
edulevel	-0.0293	0.441	-0.0278	0.456	-0.0220	0.64
Water shortage cognitive level	-0.156***	0	-0.129**	0.003	-0.0899*	0.094
Ecological cognition level	-0.304***	0	-0.341***	0	-0.312***	0.004
Disaster information	0.0578	0.101	0.0446	0.197	0.0232	0.595
Farm ratio	-0.153	0.327	-0.0455	0.766	-0.0178	0.927
Development of water rights market	-0.404***	0	-0.253*	0.025	-0.163	0.252
Feelings of rising water prices	-0.0284	0.506	-0.0649	0.122	-0.0247	0.641
Village rain conditions	-0.179**	0.033	-0.231**	0.005	-0.276***	0.008
Village water disputes	0.242***	0	0.388***	0	0.471***	0
Village collective economic level	0.252***	0	0.169***	0	0.0785	0.108
_cons	6.113***	0	6.252***	0	6.551***	0
	10muabove N = 106					
	q25	P-value	q50	P-value	q75	P-value
Restrictive measure	-0.0142	0.858	-0.0707	0.433	-0.0421	0.722
Incentive measures	-0.128	0.205	-0.0624	0.585	-0.0423	0.778
Guided measures	-0.482***	0	-0.261*	0.064	-0.228	0.215
agelevel	0.0313	0.845	-0.00623	0.973	0.0617	0.796
gender	-0.192	0.382	0.0307	0.902	-0.105	0.747
edulevel	0.106	0.22	0.116	0.239	0.0989	0.442
Water shortage cognitive level	-0.165*	0.087	-0.154	0.159	0.0202	0.888
Ecological cognition level	-0.862***	0	-0.503**^8^	0.009	-0.0857	0.73
Disaster information	-0.203**	0.036	-0.0864	0.429	-0.0983	0.493
Farm ratio	-0.0340	0.927	0.0523	0.901	-0.180	0.745
Development of water rights market	-1.280***	0	-0.902**^8^	0.003	-0.324	0.399
Feelings of rising water prices	-0.0880	0.266	-0.115	0.2	-0.0417	0.723
Village rain conditions	-0.366	0.086	-0.175	0.467	0.160	0.613
Village water disputes	0.362***	0	0.450***	0	0.518***	0
Village collective economic level	0.0579	0.53	0.0749	0.475	0.0635	0.644
_cons	9.377***	0	7.058***	0	4.801*^8^	0.012

Note

***, **, * indicate significance levels of 1%, 5%, and 10%, respectively.

The impact of different types of interventions on scale heterogeneous farmers is discussed first. As can be seen from Table 3, the incentive measures passed the significance level test in the samples of (0,5), (5,10), and the sign is negative, indicating that the incentive measures are effective for farmer households with a cultivated land size of 10 mu or less. The effect is obvious. According to the size of the coefficient, it can be judged that as the size of the farmers increases, the incentive effect gradually weakens. When the scale of operation reaches 10 acres or more, the incentive measures fail. The economic compensation cannot make up for the cost loss caused by reducing the production of groundwater. In order to compete for limited groundwater resources, large-scale farmers use high resource fusion capabilities to mine groundwater. The guided measures have passed the significance in samples of farmers of different sizes. The level test indicates that the guidance measures can effectively control the over-exploitation of groundwater by farmers, and with the expansion of the farmer’s cultivated land scale, the guidance measures gradually replace the incentive measures, and the governance effect is more significant. By comparing the absolute values of the coefficients, it can be concluded that the measures have a great impact on farmers whose farmland scale is less than 5 acres. The guiding and incentive measures are the same. It has an impact on farmers with land scale of 5 to 10 acres, and the guiding measures have obvious effects on the governance of farmers over 10 acres. Therefore, there must be flexibility in the choice of policy tools for groundwater over-exploitation management, and small farmers focus on economic incentives from subsidies, large-scale farming households focus on education and training.

Secondly, under different water consumption scenarios, the governance effects of various types of policy interventions are different. When the water consumption is at the quantile of 0.25, the governance effect of incentive measures is the most obvious, and with the increase of farmers’ water consumption, the governance effect is weakening. When the groundwater of farmers is over-exploited to a certain extent, the incentive measures have little effect on the irrigation water consumption of farmers. Incentives such as subsidies have a diminishing marginal effect. When water consumption exceeds a certain limit, the production income from over-exploitation can offset the benefits of incentives such as subsidies. At this time, relying on incentives is not feasible. Only by linking incentives such as subsidies to water users’ extraction can the government really play a role in saving water. From the coefficients of different quantiles, guided measures and incentive measures have a certain complementary effect. When water consumption is low, incentive measures play a greater role, and in the process of increasing water consumption, guided measures’ effect on groundwater over-extraction behavior has become prominent [40–42]. From this, it is inferred that under the circumstances of future land scale, intensification and groundwater over-exploitation, the government should make full use of guiding measures such as education and publicity to change farmers’ perceptions and strengthen their awareness of the dangers caused by groundwater over-exploitation.

Restrictive measures have no significant impact on groundwater irrigation volume at various sample and quantile levels. It can be seen that, in the survey area, the restrictive measures did not play the role of water control. This can also explain the government’s continuous strengthening of supervision measures, but the over-exploitation of groundwater in the North China Well Irrigation District has been serious. The government has failed in restrictive measures. Because the restrictive measures are mainly administrative management and control, and the layout of the wells for the groundwater used by farmers is scattered, with poor monitoring and strong randomness, it is difficult for the restrictive measures to play their roles. The investigation found that the cost of privately drilling wells in some North China well irrigation areas is relatively low, and the phenomenon of privately drilling wells is common.

### Brief conclusions and policy implications

#### Brief conclusions and potential future research directions

This paper evaluates the effectiveness of various measures by selecting the North China Well Irrigation District as a typical survey area, using household survey data from 1122 households, using a quantitative analysis model, and discussing the three types of measures to restrict the overexploitation of groundwater for farmers based on the perspective of scale heterogeneity. Conclusions are as follows.

First, the governance effect of the three types of policy measures is that the guidance type is better than the incentive type and the restrictive type, and the restrictive type effect is not obvious. With the increase of the scale of farmers, the incentive effect is weakening, the reduction effect of the guidance measures is gradually strengthened, the effect of the incentive measures on the over-exploitation of groundwater for small-scale farmers is obvious, and the guidance measures and incentive measures are the same at the scale of operation (5,10). Guided measures have a significant effect on controlling over-exploitation of farmers over 10 acres.

Secondly, when the water consumption is at the quantile of 0.25, the effect of incentive measures is most obvious, and with the increase of farmers’ water consumption, the effect of guided measures is more obvious. Restrictive measures govern failure.

Thirdly, farmers’ irrigation water consumption is also affected by gender, water scarcity awareness level, ecological awareness level, disaster information acquisition capacity, famer ratio, water right market development level, water price rise feelings, village rain conditions, village water disputes, and village collective economy level. And the impact of these variables on farmers of different farmland sizes is somewhat different.

In this study, the farmers in the North China Well Irrigation District were taken as the survey objects. The samples are regional in nature and it is difficult to fully represent the situation of groundwater treatment in the country. The model may have endogenous problems caused by missing variables, but this problem cannot be solved due to the limitation of the investigation conditions. And due to the limitation of space and data, this article does not verify the impact and changes of government interventions on groundwater use behavior of farmers under different resource endowment conditions (such as land conditions, climate conditions, etc.), and then comprehensively evaluate the effectiveness of government interventions. This is also potential future research directions.

#### Policy implications

There is a "tragedy of the commons" in groundwater management, and relying on the government’s reasonable intervention is an important way to solve the problem of groundwater over-exploitation in the North China Well Irrigation District. The choice of government intervention measures must attach great importance to the heterogeneity of farmers and adjust flexibly according to different situations. Formulate groundwater resource management policies for different types of farmland households.

On the one hand, it is necessary to actively exert the governance effects of guiding and incentive measures, focusing on subjects of different sizes. For example, for farmers with a cultivated land size of more than 10 acres, they mainly rely on guided measures to restrict their groundwater over-exploitation behaviors. The focus is to increase publicity and education on their groundwater regulation behaviors from multiple perspectives, such as water shortages, ecological environmental protection, and disaster information acquisition. For small farmers under 5 acres, the government should use incentive measures to mobilize their enthusiasm for water conservation, increase water-saving subsidies and price incentives, and formulate reasonable subsidies and incentives with practical incentive effects based on the cost-benefit expectations of small farmers. On the other hand, farmers are encouraged to report water consumption by themselves, or use metered irrigation tools such as IC cards (irrigation metering controller, which is similar with mobile card), and use digital technology and remote sensing technology to establish and improve data monitoring and early warning systems for groundwater irrigation of rural households, and based on groundwater consumption of heterogeneous farmers. Need to implement point-to-point dynamic supervision, set differentiated water consumption warning red lines, and implement targeted dynamic adjustments to incentive, guidance and constraint policy interventions based on data results and changes in soil conditions.

In addition, attention should also be paid to the establishment of a water rights market, the cultivation of intensive scale operation farmers, and the optimization of the village water environment, and to avoid overexploitation of groundwater and waste of water resources while expanding the village’s economic development and small farmers’ transition to moderate scale operations. Serious consequences, eliminating the hidden dangers of groundwater over-exploitation in the process of rural development and construction.

## Supporting information

S1 Table All the variables data of the survey sample. Note: dmu indicates the sample number, id indicates the different village, other varibles’ meanings were listed in the Table 1.
(XLSX)

## References

[1] Holst J., Liu W., Zhang Q., and Doluschitz R. Crop evapotranspiration, arable cropping systems and water sustainability in southern Hebei, P.R. China. *Agricultural Water Management*, 2014, 47–54.

[2] Zhang L, Heerink N, Dries L, et al. Water users associations and irrigation water productivity in northern China, *Ecological Economics*, 2013, no.95: 128–136.

[3] Li Ming-Guang et al. Effects of groundwater exploitation and recharge on land subsidence and infrastructure settlement patterns in Shanghai.” Engineering Geology 2021(282): 105995.

[4] Rithi A T, Antara Banerjee, Abhijit Mitra, Keerthi Nethaji, Divya Ilanchoorian, Radhakrishnan Arun Kumar, A concise review of the impact of groundwater pollution in coastal regions on human gut microbiome composition and its effect on human health,Groundwater for Sustainable Development, 2024,(26)101187,ISSN 2352-801X, 10.1016/j.gsd.2024.101187.

[5] Mohan C., Gleeson T., Forstner T., Famiglietti J. S., & de Graaf I. Quantifying groundwater’s contribution to regional environmental-flows in diverse hydrologic landscapes. Water Resources Research, 2023, 59, e2022WR033153. 10.1029/2022WR033153

[6] Crespo Daniel, Albiac Jose, Dinar Ariel, Esteban Encarna, Kahil Taher. Integrating ecosystem benefits for sustainable water allocation in hydroeconomic modeling.PLOS ONE 2022. doi: 10.1371/journal.pone.0267439 35511815 PMC9070880

[7] Zhilai Zheng. Policy Factors and Water Saving Behavior of Agricultural Water Users. *Journal of South China* Agricultural University(Social Science Edition), 2013,no.12:27–33.

[8] Chen S., Wang Y., Zhu T. Exploring China’s farmer-level water-saving mechanisms: analysis of an experiment conducted in Taocheng District, Hebei Province. *Water*,. 2014.,no.6: 547 (Switzerland)

[9] Ying Liu, Jikun Huang, Jinxia Wang. Impact of water price policy on irrigation water use and planting income.*China Economic Quarterly*, 2015,no.14: 1375–1392.

[10] Minjun Shi, Lei Wang, Xiaojun Wang. Water supply and demand pattern change and driving factors in Zhangye City after Heihe River watershed.*Resources Science*, 2011,no.33:1489–1497.

[11] Yahua Wang, Jiazhe Wu, Guangheng Ni. International Experience of Water Education and Its Enlightenment to China.*China Water Resources*, 2016,no.16:4–9+17.

[12] Tao Xu, Minjuan Zhao, Erhui Li, Dan Qiao, Qian Lu. The continuous adoption of large-scale operation and farmers’ "two types of technology"——Taking the drip irrigation technology of Minqin County as an example.*Journal of Arid Land Resources and Environment*, 2018, no.32:37–43.

[13] Jinxia Wang, Zhigang Xu, Jikun Huang,ScottRozelle. Reform of water resources management system, agricultural production and anti-poverty.*China Economic Quarterly*, 2005,no.4:189–202.

[14] Cameron T, Wright M. Determinants of household water conservation retrofit activity: A discrete choice model using survey data.*Water Resources Royal Economic Society*, 1990,no.26:179–188.

[15] Gardner G. T., and Stern P. C. Environmental Problems and Human Behavior, Allyn and Bacon, Boston, Mass. 1996,no.56: 407–424.

[16] NiuKunyu Wu Jian. Economic Analysis of the Influence of Agricultural Irrigation Water Price on Farmers’ Water Consumption.*China Population*,*Resources and Environment*, 2010,no.20:59–64.

[17] Changshun Liu, Lijuan Du, Hongkun Wang. Comparative Study on Influencing Factors of Farmers’ Irrigation Decision Behavior.*China Water Resources*, 2018,no.19: 36–38+42.

[18] Junrui Li, Xiqin Wang, Yumeng Wang. Behavior Research of Farmers Participating in Irrigation——Taking Shijin Irrigation District of Hebei Province as an Example.*Journal of Agrotechnical Economics*, 2018,no.5:66–76.

[19] Aoyagi K, Sawada Y, Coomes O T.Irrigation infrastructure and trust: Evidence from natural and lab-in-the-field experiments in rural communities.World Development, 2022, 156.

[20] Mandal R, Maity S.Does a Diversified Crop Portfolio Make Farmers More Efficient? A Stochastic Production Frontier Analysis of Farm-level Data from Assam, India.International Journal of Rural Management, 2022(1):18. doi: 10.1177/0973005221997580

[21] Xiaojun Wang, Minjun Shi, Lei Wang. Ways to alleviate water crisis in drought-scarce areas: policy effects of water demand management.*Journal of Natural Resources*, 2013, no.28:1117–1129.

[22] Bate R. Water—can property rights and markets replace conflict? In: Morris J.(Ed.), Sustainable Development: Promoting Progress or Perpetuating Poverty? Profile Books, London.2002.

[23] Hui Ai,GuoDeen. Groundwater over-exploitation threatens the North China Plain. *Ecological Economy*. 2018.

[24] Yongsong Liao, Weizhuo, Bao ziyun, Huang Qingwen. Groundwater resources management system, status quo and consequences.*Water Resources Development Research*, 2005, no.8: 37–41.

[25] Jian Zhang, Shuyi Feng, Peixin Zhu. Will government intervention in the agricultural land transfer market exacerbate the rural internal income gap?——Based on the survey of four counties in Jiangsu Province.*Journal ofPublic Management*, 2017,no.14:104–116+158–159.

[26] Hui Wang. A Logic Study on the Choice and Application of Policy Instruments——Taking the Supply of Rural Public Products in Z Township of Sichuan as an Example.*Journal of Public Management*, 2014,no.11:14–23+139–140.

[27] Figureau A G, Montginoul M, Rinaudo J D, et al. Policy instruments for decentralized management of agricultural groundwater abstraction:: A participatory evaluation, *Ecological Economics*, 2015,no.119:147–157.

[28] Bowles S. Policies Designed for Self-Interested Citizens May Undermine "The Moral Sentiments": Evidence from Economic Experiments, *Science*, 2008,no.320:1605–1609. doi: 10.1126/science.1152110 18566278

[29] Feike T, Henseler M. Multiple Policy Instruments for Sustainable Water Management in Crop Production—A Modeling Study for the Chinese Aksu-Tarim Region, *Ecological Economics*, 2017, 42–54.

[30] Dinar A, Mody J. Irrigation water management policies: Allocation and pricing principles and implementation experience, *Natural Resources Forum*, 2004,no.28:112–122.

[31] HuJianfeng Huang Jianqiu. The Effectiveness of Water Pollution Control and Its Policy Tools: A Case Study of Shuitou Tannery Base in Pingyang County, Wenzhou City. *Management World*, 2008,no.5:77–84.

[32] Zhang jing Ma Guihong, Gao ya Shen Yanjun, Xiaoying Liu, Yan Zou, Dai Maohua Analysis on influenceing factors of groundwater level change in typical well irrigation area in piedmont plain of North China. Journal of Hohai University (Natural Science), 2022, (1),50.

[33] Zhenan Li,HuangShaoan. Institutional Failure and Technological Innovation: An Economic Analysis of Farmers Burning Straw.*China Rural Survey*, 2002, no.5:11–16+80.

[34] Jing Wang, Huancheng Pang, Tianzhi Ren et al. Fenirizing Tendency and countermeasures for science and technology propagation of water-saving agricultural in China. Journal of anhui agricultural sciences, 2009 ((13): 6301–6304.

[35] Venot J, Reddy V R, Umapathy D, et al. Coping with drought in irrigated South India: Farmers’ adjustments in NagarjunaSagar, *Agricultural Water Management*, 2010,no.97:1434–1442.

[36] Tang J, Folmer H, Xue J, et al. Estimation of awareness and perception of water scarcity among farmers in the Guanzhong Plain, China, by means of a structural equation model, *Journal of Environmental Management*, 2013,55–62. doi: 10.1016/j.jenvman.2013.03.051 23666070

[37] Ajzen I. The theory of planned behavior. *Organizational Behavior and Human Decision Processes*, 1991,no.50: 179–211.

[38] Yiming Liu, Biliang Luo. The Impact of tradable Water Rights Arrangement on Farmers’ Irrigation Water Use Behavior——Based on the Theoretical Analysis of Farmers’ Behavior Model.*Mathematics in Practice and Theory*, 2014,no.44:7–14.

[39] Varghese S. K., Veettil P. C., Speelman S., Buysse J., Huylenbroeck G. V. Estimating the causal effect of water scarcity on the groundwater use efficiency of rice farming in South India, *Ecological Economics*, 2013,no.86:55–64.

[40] Heyman Josiah M., Mayer Alex, Alger Jessica. Predictions of household water affordability under conditions of climate change, demographic growth, and fresh groundwater depletion in a southwest US city indicate increasing burdens on the poor. Plos one, 2022 10.1371/journal.pone.0277268 PMC9683544 36417475

[41] Dastkhan H., & Mohamadi G. What are the sustainable water policies in central regions of iran? an integrated water resource management model. Water Economics and Policy, 2023,09(01).

[42] Lv N., Liu F., Zhu H., & Wang G. Effect of government intervention and market incentives on farmer organic fertilizer application behavior and agricultural emission reduction. Natural hazards review. 2023.

